# Monocot and dicot MLO powdery mildew susceptibility factors are functionally conserved in spite of the evolution of class-specific molecular features

**DOI:** 10.1186/s12870-015-0639-6

**Published:** 2015-10-26

**Authors:** Michela Appiano, Domenico Catalano, Miguel Santillán Martínez, Concetta Lotti, Zheng Zheng, Richard G F Visser, Luigi Ricciardi, Yuling Bai, Stefano Pavan

**Affiliations:** Wageningen UR Plant Breeding, Wageningen University & Research Centre, Droevendaalsesteeg 1, 6708 PB Wageningen, The Netherlands; Institute of Biosciences and Bioresources, Italian National Research Council, via Amendola 165/A, 70126 Bari, Italy; Department of Agricultural, Food and Environmental Sciences, University of Foggia, Via Napoli 25, 71100 Foggia, Italy; Institute of Vegetables and Flowers, Chinese Academy of Agricultural Sciences, No. 12 Zhongguan Cun Nan Da Jie, 100081 Beijing, China; Department of Soil, Plant and Food Science, Section of Genetics and Plant Breeding, University of Bari, Via Amendola 165/A, 70126 Bari, Italy

**Keywords:** MLO, Powdery mildew, Angiosperms, Evolution, Plant breeding

## Abstract

**Background:**

Specific members of the plant Mildew Locus O (MLO) protein family act as susceptibility factors towards powdery mildew (PM), a worldwide-spread fungal disease threatening many cultivated species. Previous studies indicated that monocot and dicot MLO susceptibility proteins are phylogenetically divergent.

**Methods:**

A bioinformatic approach was followed to study the type of evolution of Angiosperm MLO susceptibility proteins. Transgenic complementation tests were performed for functional analysis.

**Results:**

Our results show that monocot and dicot MLO susceptibility proteins evolved class-specific conservation patterns. Many of them appear to be the result of negative selection and thus are likely to provide an adaptive value. We also tested whether different molecular features between monocot and dicot MLO proteins are specifically required by PM fungal species to cause pathogenesis. To this aim, we transformed a tomato mutant impaired for the endogenous *SlMLO1* gene, and therefore resistant to the tomato PM species *Oidium neolycopersici*, with heterologous *MLO* susceptibility genes from the monocot barley and the dicot pea. In both cases, we observed restoration of PM symptoms. Finally, through histological observations, we demonstrate that both monocot and dicot susceptibility alleles of the *MLO* genes predispose to penetration of a non-adapted PM fungal species in plant epidermal cells.

**Conclusions:**

With this study, we provide insights on the evolution and function of *MLO* genes involved in the interaction with PM fungi. With respect to breeding research, we show that transgenic complementation assays involving phylogenetically distant plant species can be used for the characterization of novel *MLO* susceptibility genes. Moreover, we provide an overview of MLO protein molecular features predicted to play a major role in PM susceptibility. These represent ideal targets for future approaches of reverse genetics, addressed to the selection of loss-of-function resistant mutants in cultivated species.

**Electronic supplementary material:**

The online version of this article (doi:10.1186/s12870-015-0639-6) contains supplementary material, which is available to authorized users.

## Background

The plant *Mildew Locus O* (*MLO*) gene family codes for proteins harboring seven transmembrane domains and a calmodulin-binding site, topologically reminiscent of metazoan and fungal G-protein coupled receptors (GPCRs) [1]. Following the completion of plant genome sequencing projects, a number of homologs varying from 12 to 19 has been identified in the *MLO* gene families of diploid species, namely Arabidopsis, rice, grapevine, cucumber, peach, woodland strawberry and sorghum [1–6].

Specific homologs of the *MLO* gene family act as susceptibility factors towards fungi causing the powdery mildew (PM) disease, worldwide spread and causing severe losses in agricultural settings. Inactivation of these genes, through loss-of function mutations or silencing, indeed results in resistance (referred to as *mlo-*based resistance) in several plant species [7]. The first *MLO* gene described as required for PM pathogenesis was barley *HvMLO* [8, 9]. Since then, *MLO* susceptibility genes have been functionally characterized in rice (*OsMLO3)*, wheat (*TaMLO_A1* and *TaMLO_B1*)*,* Arabidopsis (*AtMLO2*, *AtMLO6* and *AtMLO12*), tomato (*SlMLO1*), pepper (*CaMLO2*), tobacco (*NtMLO1*), pea (*PsMLO1*), lotus (*LjMLO1*) and barrel clover (*MtMLO1*) [10–17].

Defense mechanisms involved in *mlo*-based resistance prevent fungal penetration in epidermal cells and are associated with the formation of cell wall appositions, referred to as papillae [11]. Similar pre-penetration defense measures also take place in non-host resistance, following the interaction between PM fungal species and plant species beyond their host range. Consistent with the hypothesis of involvement of *MLO* genes in non-host resistance, loss of function of *HvMLO* in the interaction between barley and the wheat PM fungus *Blumeria graminis* f. sp. *tritici* is associated with decreased rate of penetration and lower incidence of epidermal cell death, the latter being a post-penetration defense mechanism [18, 19].

Several studies have been addressed to the characterization of regions of relevance for the functionality of MLO proteins. Multiple alignments have pointed out the occurrence of residues highly conserved within the whole MLO family, which were therefore predicted to provide a common protein structural scaffold [12, 20]. In addition, the occurrence of residues and motifs specifically conserved in putative orthologs of barley HvMLO has been reported [9]. Finally, functionally important residues for MLO susceptibility proteins have been inferred by the association of naturally occurring and induced mutations with partial or complete PM resistance [11, 12, 21–25].

In our previous studies, we showed that phylogenetically related dicot *MLO* genes of the same botanic family are conserved for their function as a susceptibility gene to PM [6, 16]. Notably, monocot and dicot MLO proteins involved in PM susceptibility group in clearly separated phylogenetic clades (e.g. [2, 9]). Here, we show that the evolution of Angiosperm PM susceptibility genes led to the fixation of class-specific molecular traits. Many of them appear to be the result of negative selection. By means of transgenic complementation assays, we demonstrate that, despite having different conservation patterns, monocot and dicot *MLO* susceptibility genes are essentially conserved with respect to functional features having a role in interactions with PM fungi. Consequences of our findings for plant breeding research are discussed.

## Results

### Class-specific molecular features of Angiosperm MLO homologs required for PM susceptibility

**Fig. 1:**
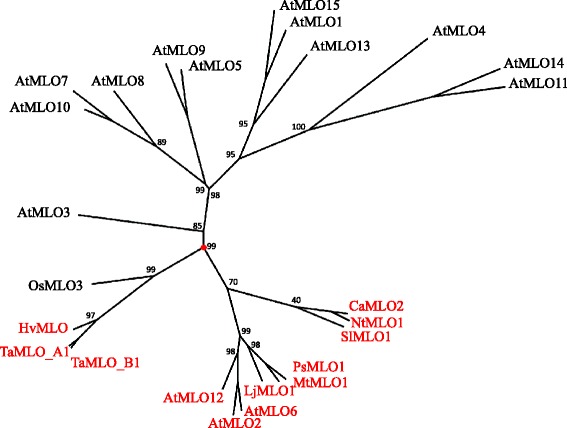
Unrooted radial phylogenetic tree of MLO powdery mildew susceptibility proteins. The tree includes, in red, all the monocot and dicot MLO homologs shown to be required for powdery mildew susceptibility (Arabidopsis AtMLO2, AtMLO6 and AtMLO12, tomato SlMLO1, pepper CaMLO2, tobacco NtMLO1, pea PsMLO1, lotus LjMLO1, barrel clover MtMLO1, barley HvMLO, wheat TaMLO_B1 and TaMLO_A1b and rice OsMLO3), and the remaining homologs of the Arabidopsis AtMLO family. Numbers at each node represent bootstrap support values (out of 100 replicates)

**Fig. 2:**
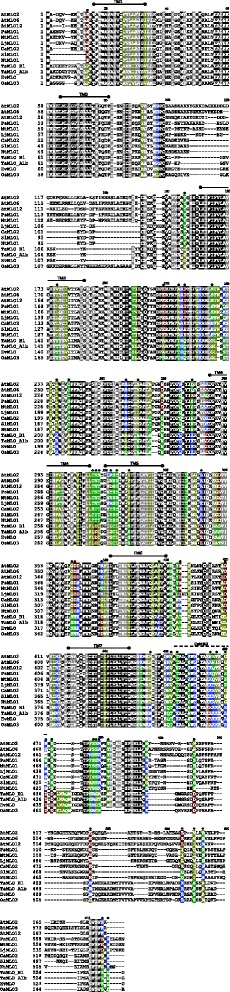
Multiple alignment of MLO powdery mildew susceptibility proteins. The dataset is composed of all the monocot (barley HvMLO, rice OsMLO3, wheat TaMLO_B1 and TaMLO_A1b), and dicot (Arabidopsis AtMLO2, AtMLO6 and AtMLO12, tomato SlMLO1, pepper CaMLO2, tobacco NtMLO1, pea PsMLO1, lotus LjMLO1 and barrel clover MtMLO1) MLO homologs shown to act as powdery mildew susceptibility factors. The positions of the seven MLO transmembrane domains (TM1-TM7) and the calmodulin binding domain (CaMBD) are identical to the ones reported by Feechan *et al.* [2], *Functional Plant Biology*, 35: 1255–1266. Black color indicates alignment positions in which invariable residues are present. Grey color indicates alignment positions which do not contain class-specific residues and are conserved with respect to biochemical properties. Other colors indicate alignment positions in which there are class-specific residues in monocots, dicots, or both: yellow indicates hydrophobic residues (G, A, V, L, I, F, W, M, P); blue indicates polar basic residues (K,R,H); red indicates polar acidic residues (D, E); green indicates polar uncharged residues (S, T, C, Y, N, Q). Black dots highlight 44 alignment positions in which class-specific residues are substituted in the other class by residue(s) having different biochemical properties

Previous studies indicated that dicot and monocot MLO proteins with a putative or ascertained role in susceptibility to PM fungi group in two different phylogenetic clades (e.g. [2, 9]). This was confirmed by performing a new UPGMA-based phylogenetic analysis involving all the 12 MLO homologs which have been until recently functionally related to PM susceptibility (Fig. 1). Aiming to detect molecular features responsible for such phylogenetic divergence, the same MLO homologs were used as dataset for protein multiple alignment (Fig. 2). Notably, this led to the identification of 41 alignment positions in which residues invariable throughout dicots are absent in monocots, and 84 alignment positions in which residues invariable throughout monocots are absent in dicots. In 44 alignment positions, class-specific residues are replaced in the other class with residues having different properties, according to the chemical features of their side-chain group (hydrophobic, polar basic, polar acidic and polar uncharged).

### Adaptive relevance of class-specific molecular features supported by evolutionary analysis

In order to make inference on the evolutionary events leading to the above mentioned class-specific molecular features, we performed a codon-based Single-Likelihood Ancestor Counting (SLAC) analysis on the difference of nonsynonymous to synonymous substitutions per nonsynonymous and synonymous sites (dN-dS). Tests were conducted to predict the evolution of each codon: neutral/dN = dS or negative (purifying)/dN < dS. We decided to restrict the analysis to a panel of nine dicot *MLO* susceptibility genes, as only four monocot *MLO* homologs have been so far associated with PM pathogenesis and the dN-dS analysis can provide significant results only when using a sequence dataset which is not too small. We found 130 codons under significant negative selection, coding for amino acids scattered throughout MLO protein domains. Among the 130 codons, 27 are translated into class-specific residues, which are therefore predicted to provide an adaptive value (Additional file 1).

### Functional conservation of monocot and dicot MLO susceptibility genes

**Fig. 3:**
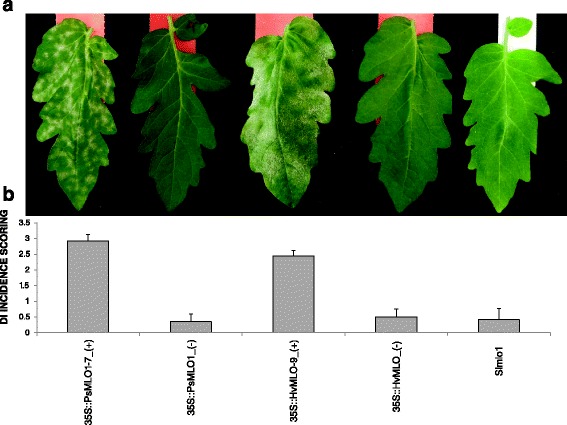
Transgenic overexpression of pea *PsMLO1* and barley *HvMLO* in the tomato mutant line Slmlo1. Panel **a** shows the phenotypes of two selected individuals of the T_2_ family 35S::*PsMLO1*-7, segregating for the presence (first from the left) or the absence (second from the left) of the transgene, two selected individuals of the T_2_ family 35S::*HvMLO*-9, segregating for the presence (third from the left) or the absence (second from the right) of the transgene, and one individual of the Slmlo1 line (first from the right), in response to the tomato powdery mildew fungus *Oidium neolycopersici*. Panel **b** from left to right shows average disease index (DI) values relative to transgenic plants (+) of the 35S::*PsMLO1*-7 T_2_ family, non-transgenic plants (−) of three T_2_ families segregating for the 35S::*PsMLO1* construct, transgenic plants of the 35S::*HvMLO*-9 T_2_ family, non-transgenic plants of three T_2_ families segregating for the 35S::*HvMLO* construct and the Slmlo1 line. Standard deviation bars refer to six 35S::*PsMLO1_(+)* individuals, nine 35S::*HvMLO_(+)* individuals, 7 *PsMLO1*_(−) individuals, 7 *HvMLO*_(−) individuals and 10 Slmlo1 individuals

We tested whether different molecular features between monocot and dicot MLO proteins are specifically required by PM fungal species infecting either one or the other class of Angiosperms. To this aim, we developed two constructs for the transgenic expression of a monocot (barley *HvMLO*) and a dicot (pea *PsMLO1*) *MLO* gene in the tomato Slmlo1 line, which is homozygous for a loss-of-function mutation in the endogenous gene *SlMLO1* and therefore resistant to the tomato PM fungus *Oidium neolycopersici*. We reasoned that complementation and restoration of PM symptoms would have occurred only in case of functional conservation between *SlMLO1* and any of the two tested transgenes. In total, nineteen 35S::*PsMLO1* and 20 35S::*HvMLO* transformants were obtained. In both cases, 18 individuals were obtained showing variable transgene expression levels. For each construct, three T_1_ plants displaying high transgene expression (35S::*PsMLO1*-4,−6 and−7 and 35S::*HvMLO*-9,−10 and−15) were self-pollinated to generate T_2_ families (Additional file 2 ). Ten individuals from each T_2_ family were tested for the presence or the absence of the transgene and challenged with *O. neolycopersici*. Transgenic individuals of the three T_2_ families overexpressing *PsMLO1* (35S::*PsMLO1_*(+)) displayed PM symptoms with an average D.I. (disease index) score ranging from 2.87 to 2.92. Transgenic individuals of the three T_2_ families overexpressing *HvMLO* (35S::*HvMLO*_(+)) showed an average D.I. score ranging from 1.8 to 2.4. In contrast, all non-transgenic 35S::*PsMLO1_*(−) and 35S::*HvMLO*_(−) T_2_ individuals displayed, similar to the Slmlo1 plants, hardly any fungal growth (Fig. 3 and Additional file 3). For transgenic plants of the three 35S::*HvMLO* T_2_ families, positive correlation was found between average D.I. and transgene expression level of corresponding T_1_ plants (Fig. 3 and Additional file 2 and 3). Together, these results indicate that monocot and dicot *MLO* susceptibility genes are functionally conserved with respect to molecular features required for PM pathogenesis.

### Functional conservation of monocot and dicot MLO susceptibility genes in non-host interactions

**Fig. 4:**
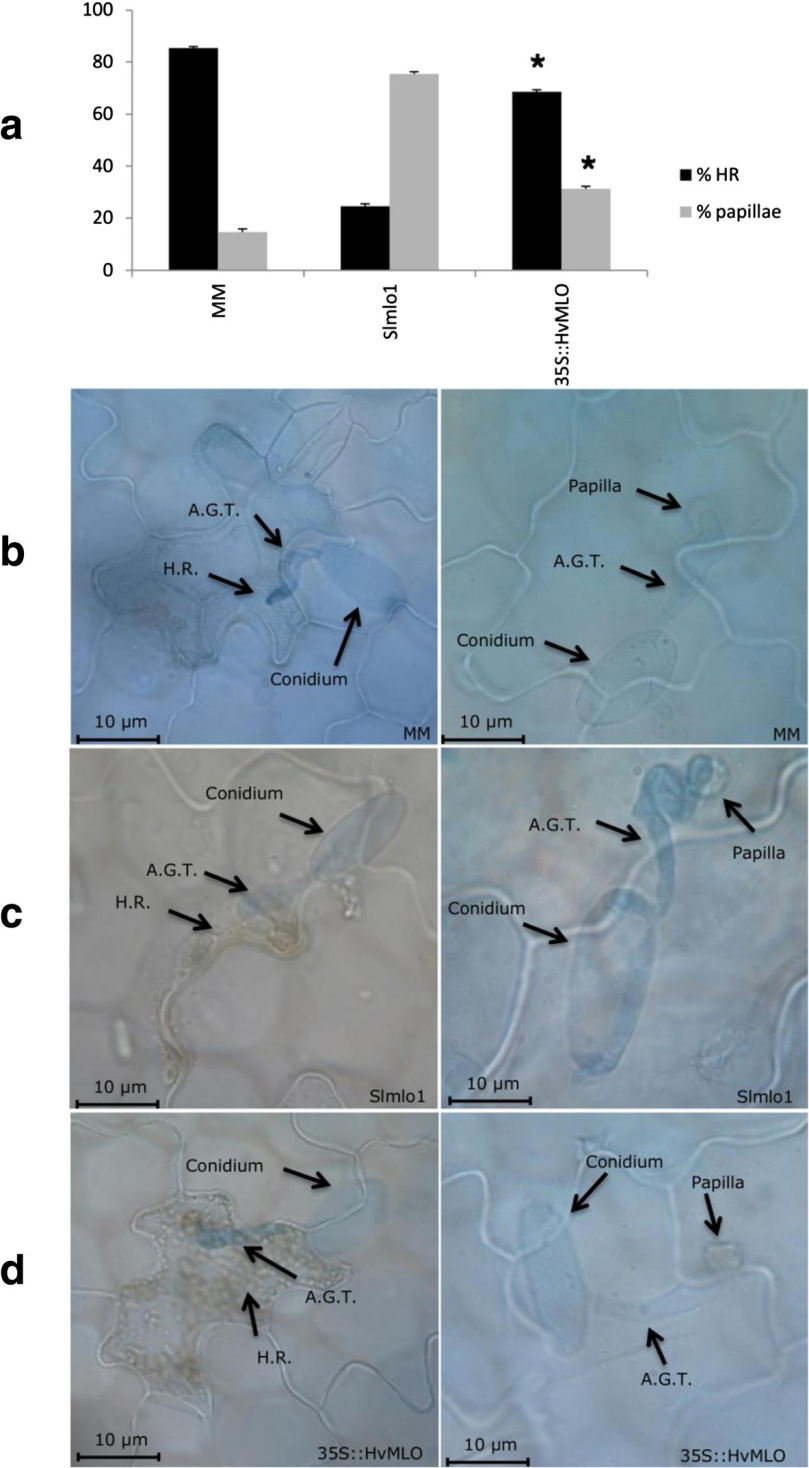
Functional conservation of *SlMLO1* and *HvMLO* in the tomato/*Blumeria graminis* f.sp. *hordei* (*Bgh*) interaction. Panel **a** shows the ratio of penetrated and non-penetrated epidermal cells, assessed in function of infection units showing hypersensitive response (H.R.) and papillae, respectively, in the following genotypes: the *mlo* mutant line Slmlo1; the cultivar MM, with a similar genetic background and carrying wild-type *SlMLO1*; transgenic plants of a T_2_ family overexpressing barley *HvMLO* in the Slmlo1 genetic background (35S::*HvMLO*-9). Panel **b**, **c** and **d** show, in the same genotypes, fungal structures (conidiospore and appressorium germination tube -A.G.T.-) and cellular events (the formation of papillae and H.R.) arresting fungal growth before and after penetration, respectively

We next investigated whether functional conservation between monocot and dicot MLO homologs also holds true in non-host plant-PM interactions. To this aim, we used the PM species *B. graminis* f.sp. *hordei* (*Bgh*) to inoculate plants of the Slmlo1 mutant line, the cultivar Moneymaker (MM), carrying wild-type *SlMLO1*, and two of the 35S::*HvMLO* T_2_ families (35S::*HvMLO*-9 and −10, previously described in Fig. 3, Additional file 2 and 3). *Bgh* is an adapted PM on barley and a non-adapted PM to tomato. In the Slmlo1 line, 75.4 % of infection units were associated with papilla formation and 24.6 % with cell death response (Fig. 4). Compared with the Slmlo1 line, transgenic 35S::*HvMLO*-9 T_2_ plants displayed a lower level of papilla formation (31.3 %) and a higher level of cell death response (68.7 %). In MM, papilla formation and cell death occurred at a rate similar to the one in 35S::*HvMLO*-9 plants (14.6 % and 85.4 %, respectively). Taken together, this body of evidence indicates that both *HvMLO* and *SlMLO1* predispose to the penetration of a non-host pathogen.

## Discussion

The functional characterization of *MLO* homologs involved in PM susceptibility is of great interest for basic research on plant-microbe interactions as well as for plant breeding, as loss-of-function genotypes could be conveniently used to introduce durable and broad-spectrum resistance in cultivated species [7]. Results of previous investigations indicated that *mlo*-based resistance in a certain plant species can be lost by the heterologous expression of *MLO* susceptibility genes from related species of the same botanical family. Indeed, restored susceptibility has been observed in barley *HvMLO* mutants transformed with wheat *TaMLO_B1* and rice *OsMLO3,* as well as in pea *PsMLO1* mutants expressing lotus *LjMLO1* or barrel clover *MtMLO1*[12, 13]. Recently, similar evidence was shown on tomato *SlMLO1* mutants transformed with pepper *CaMLO2* or tobacco *NtMLO1* [16, 17]. Here, we investigated whether complementation can also occur by transferring *MLO* genes from more evolutionary divergent plant species. We found that, in a tomato *mlo* mutant background, transgenic expression of a *MLO* susceptibility gene from pea (a distantly related dicot species) and barley (a monocot species) is sufficient to re-establish PM susceptibility (Fig. 3 and Additional file 3). This finding indicates that, despite phylogenetic distance and the evolution of peculiar molecular traits (Fig. 1 and 2), monocot and dicot MLO proteins are essentially conserved with respect to features involved in the interaction with PM pathogens. In support of this conclusion, we show that the monocot gene *HvMLO* and the dicot gene *SlMLO1* both enhance penetration of the non-adapted pathogen *B. graminis* f.sp. *hordei* compared to a tomato *mlo*-mutant (Fig. 4). Moreover, after reviewing scientific literature, we found that only one out of thirty *MLO* protein substitutions so far associated with PM resistance involves a class-specific residue (a monocot-specific alanine residue in position 350 of the alignment in Fig. 2) (Table 1) [22]. The same residue is replaced in dicots by a glycine (sharing similar non-polar chemical properties of alanine, Table 1), indicating that, in this case, class-specific conservations are not associated with important changes in protein structure or function.

**Table 1:** Amino acid residues in dicot AtMLO2 and monocot HvMLO whose mutation has been associated with PM resistanc**e**. For each amino-acid, localization in any of the MLO protein domains, including seven transmembrane (TM) regions, three extracellular loops (E), three intracellular (I) loops, the N-terminus and the C-terminus, is indicated

	ᅟ

Numbers adjacent to each amino acid indicate their position in either HvMLO or AtMLO2 proteins

Barley and Arabidopsis residues in the same row correspond to each other in HvMLO/AtMLO2 protein alignment

Percentage of conservation is calculated based on the alignment of 13 MLO proteins functionally associated with powdery mildew susceptibility (AtMLO2, AtMLO6, AtMLO12, SlMLO1, CaMLO2, NtMLO1, PsMLO1, LjMLO1, MtMLO1, TaMLO_A1b, TaMLO_B1, OsMLO3 and HvMLO)

Amino acid color is according to its chemical properties: non-polar (yellow), polar, uncharged (green), polar, acidic (red), polar, basic (blue)

^a^partial resistance observed in barley, ^b^full resistance observed in Arabidopsis

We cannot exclude that class-specific traits might have minor effects on interactions with PM fungi. Indeed, by comparing three independent T_2_ families for each construct, we found that that overexpression of *PsMLO1* results in higher D.I. index scores than the one of *HvMLO* (Fig. 3 and Additional file 3). Clearly, complementation tests with several other monocot and dicot transgenes could help to answer this question.

Through the analysis of the dN-dS difference, we provide evidence for negative selection acting on several class-specific residues, which are thus likely to play a major adaptive role (Additional file 1). However, as mentioned before, transgenic complementation tests indicate that these class-specific residues are not crucial for the outcome of the interaction between plants and PM pathogens. Possibly, some of the class-specific residues identified in this study might underlie roles which are not related with the interaction with PM fungi. The implication of MLO susceptibility proteins in other physiological processes would explain why, in spite of being required for pathogenesis, they have been not excluded by evolution. With this respect, it is worth to mention that PM resistance in Arabidopsis and barley *mlo* mutants has been associated with the induction of leaf senescence, a pleiotropic phenotype [11].

We show that *MLO* homologs required for PM pathogenesis can complement a *mlo* mutant background in transgenic assays, irrespective of the phylogenetic distance between the donor and the recipient species (Fig. 3). This would be of great advantage in order to test the function of candidate *MLO* susceptibility genes which are currently being identified by several authors across cultivated species [4, 5]. Moreover, we provide an overview of MLO protein regions which are under negative selection and thus are expected to be of functional relevance. These regions represent ideal targets to select loss-of-function mutants resistant to the PM disease. With this respect, breeders may apply diverse tools, such as conventional targeted mutagenesis approaches of TILLING (targeted induced local lesions in genomes) or advanced technologies of genome editing, based on zinc finger nucleases (ZFNs), clustered regularly interspaced short palindromic repeat (CRISPR) and transcription activator-like effector nucleases (TALEN) [26–28].

## Conclusion

This work provides insights on the evolution and function of Angiosperm *MLO* susceptibility genes. We show that complementation assays similar to those carried out in this study are suitable for future activities aimed at the characterization of novel PM susceptibility factors across cultivated species. Moreover, we indicate a series of gene targets for the selection of loss-of-function *ml*o resistant mutants.

## Methods

### Bioinformatic analyses

The following MLO proteins, experimentally shown to be required for PM susceptibility, were used as dataset for CLUSTAL alignment using the CLC sequence viewer software (http://www.clcbio.com/products/clc-sequence-viewer/): Arabidopsis AtMLO2 [GenBank: NP172598], AtMLO6 [GeneBank: NP176350] and AtMLO12 [GeneBank: NP565902], tomato SlMLO1 [GeneBank: NP001234814], pea PsMLO1 [GeneBank: ACO07297], pepper CaMLO2 [GeneBank: AFH68055], lotus LjMLO1 [GeneBank: AAX77015], barrel clover MtMLO1 [GeneBank: ADV40949], barley HvMLO [GeneBank: P93766], rice OsMLO3 [GeneBank: AAK94907], wheat TaMLO_B1 [GeneBank: AAK94904] and TaMLO_A1b [GeneBank: AAK94905]. The alignment was given to Geneious v8 software (http://www.geneious.com, [29] ), to highlight amino acids with different polarity, and the online web service Phylogeny.fr (http://www.phylogeny.fr/) to construct an unrooted radial phylogenetic tree.

In order to make predictions on the type of evolution (negative or neutral) of class-specific molecular features, all the above mentioned dicot *MLO* susceptibility genes were used as dataset for a codon-based evolutionary analysis based on the difference of nonsynonymous-to-synonymous substitutions per nonsynonymous and synonymous sites (dN/dS). This was performed by using the Single-likelihood Ancestor Counting (SLAC) method implemented by the Datamonkey web server (www.datamonkey.org). The default p-value of 0.1 was taken as threshold to call codons under significant negative selection.

### Isolation and cloning of full-length PsMLO1 and HvMLO

Total RNAs from pea (cultivar Sprinter) and barley (cultivar Maythorpe) were isolated by using the RNeasy plant mini kit (Qiagen), and corresponding cDNAs were synthesized by using the SuperScript III first-strand synthesis kit (Invitrogen) and the oligo(dT)_20_ primer. Specific primer pairs, named PsMLO1-Fw/PsMLO1-Rev and HvMLO-Fw/HvMLO-Rev (Additional file 4: Table S2) were manually designed in order to amplify the *PsMLO1* and *HvMLO* full-length coding sequences, respectively. PCR reactions were performed by using the high-fidelity Phusion DNA polymerase (New England Biolabs) and an annealing temperature of 55 °C. Amplicons were ligated into the Gateway-compatible vector pENTR D-TOPO (Invitrogen) and cloned into the *E. coli* One Shot® TOP10 cells (Invitrogen), according to the manufacturer’s instructions. After selecting positive colonies by colony PCR, using the two gene-specific primer pairs above mentioned, recombinant plasmids were extracted and their inserts were sequenced. A single colony for each construct was selected, in which the inserts resulted to have sequences identical to those of *HvMLO* and *PsMLO1* deposited in the NCBI database.

### Generation and functional characterization of transgenic SlMLO1 mutant tomato plants expressing PsMLO1 and HvMLO

Following the manufacturer instructions (Invitrogen), cloned *HvMLO* and *PsMLO1* gene sequences were inserted by LR recombination into the binary plasmid vector pK7WG2, which harbors the 35S Cauliflower Mosaic Virus (CaMV) promoter and the marker gene *nptII* for kanamycin resistance selection. Plasmids were then transferred to *E. coli* and positive colonies were screened by colony PCR and sequencing, as previously mentioned. Finally, recombinant vectors were extracted and transferred to the AGL1-*vir*G strain of *A. tumefaciens* by electroporation.

The transformation of the tomato *ol-2* mutant line, carrying a loss-of-function mutation of the PM susceptibility gene *SlMLO1*, was performed according to the methods described by [6] and [16]. The evaluation of the expression levels of *PsMLO1* and *HvMLO* in T_1_ plants was carried out by real-time qPCR using the primer pairs qPsMLO1-Fw/qPsMLO1-Rev and qHvMLO-Fw/qHvMLO-Rev (Additional file 4). A primer pair designed on the *elongation factor 1α* gene (qEF-Fw/qEF-Rev) was used for relative quantification (Additional file 4).

### Functional characterization of host and non-host interactions

For each of the two transgenes above mentioned, three T_1_ individuals showing the highest expression levels were allowed to self-pollinate, resulting in a total of six T_2_ families. Individuals of each family were assayed for the presence/absence of the overexpression construct by means of PCR, using the primer pairs NPTII_Fw/ NPTII_Rev and 35S-Fw/35S-Rev designed on the nptII marker gene and the 35S promoter, respectively (Additional file 4). Ten resistant Slmlo1 plants carrying the loss-of-function *SlMLO1* allele and ten individuals of each family were challenged with an isolate of the tomato PM fungus *O. neolycopersici* maintained at the Plant Breeding Department of the University of Wageningen, The Netherlands. Inoculation was performed as described by [30], spraying 4 weeks-old plants with a suspension of conidiospores obtained from freshly sporulating leaves of heavily infected plants and adjusted to a final concentration of 4 × 10^4^ spores/ml. Inoculated plants were grown in a greenhouse compartment at 20 ± 2 °C with 70 ± 15 % relative humidity. Disease evaluation was visually carried out 15 days after inoculation, based on the presence of disease signs on the third and fourth leaf, according to the scale from 0 to 3 reported by [10].

For the functional characterization of a non-host interaction, seeds from one of the three 35S::*HvMLO* T_2_ families previously tested were surface-sterilized and sown on half-strength Murashige and Skoog (MS) agar supplemented with 50 mg/ml kanamycin for selection of transgenic plants. Seeds were left for 2 days at 4 °C and then transferred to a growing chamber for 10 days. Five transgenic seedlings were transplanted in pots and transferred to a greenhouse compartment. Three barley plants of the PM susceptible cultivar Manchuria, five Slmlo1 plants and five MoneyMaker plants were used as controls. An isolate of *B. graminis* f. sp. hordei (*Bgh*) collected in Wageningen (Wag.04) was used for the inoculation. This was performed by rubbing Manchuria leaves heavily infected with *Bgh* on the third tomato leaf. After 72 h, in which inoculated plants were kept in a climate chamber at 20 °C, 16 h of light/day and 70 % RH, a 4 cm^2^ segment was cut from the inoculated leaves (third leaf). Three samples were taken from 3 plants of each genotype.

Each leaf segment was bleached is a 1:3 (v/v) acetic-acid/ethanol solution and 48 h later stained in 0.005 % Trypan Blue as described by [31]. The rate of fungal penetration was estimated by the frequency of infection units showing epidermal cell death. For each genotype, three biological replicates were considered, considering at least 100 infection units.

## Additional files

Additional file 1: Table S1. Codons under significant negative selection in PM susceptibility genes. Codon numbers refer to positions in the alignment of nine dicot *MLO* genes (*AtMLO2*, *AtMLO6*, *AtMLO12*, *PsMLO1*, *MtMLO1*, *LjMLO1*, *CaMLO2*, *SlMLO1*, *NtMLO1*) experimentally shown to act as powdery mildew susceptibility genes. Amino acid residues corresponding to each codon in barley HvMLO and pea PsMLO1 are indicated. For each residue, localization in any of the MLO protein domains, including seven transmembrane (TM) regions, three extracellular loops (E), three intracellular (I) loops, the N-terminus and the C-terminus, is indicated. Codons marked in bold are translated into class-specific residues. The threshold p-value was 0.1, representing the default value for Single-likelihood Ancestor Counting (SLAC) analysis implemented by the Datamonkey web server. (DOCX 30 kb) Additional file 2: Figure S1. Expression levels of *PsMLO1* and *HvMLO* after transformation*.* Panel A) and panel B) show the expression of *PsMLO1* and *HvMLO* in 19 and 20 T_1_ individuals, respectively, which were obtained by the transformation of the tomato mutant line Slmlo1, harboring a loss-of-function mutation of the endogenous *SlMLO1* gene. Asterisks indicate T_1_ individuals selected for self-pollination and the development of T_2_ families. (PDF 173 kb) Additional file 3: Figure S2. Effects of transgenic overexpression of pea *PsMLO1* and barley *HvMLO* in the tomato mutant line Slmlo1. Average disease index (DI) values and phenotypes are referred to transgenic plants of two additional T_2_ families segregating for *PsMLO1* [35S::*PsMLO1*-4 and 35S::*PsMLO1*-6, panel a) and b)] and two additional T_2_ families segregating for *HvMLO* [35S::*HvMLO*-10 and 35S::*HvMLO*-15), panel c) and d)]. Data relative to the Slmlo1 mutant line, used as genetic background for transformation, and non-transgenic plants of three T_2_ families for each overexpression construct (35S::*PsMLO1*_(−) and 35S::*HvMLO*_(−)) are also shown. (PDF 304 kb) Additional file 4: Table S2. Primer pairs used in this study. (DOCX 14 kb)
