# Supplementary material for: Monocot and dicot MLO powdery mildew susceptibility factors are functionally conserved in spite of the evolution of class-specific molecular features
Source: BMC Plant Biol. 2015 Oct 26;15:257. doi: 10.1186/s12870-015-0639-6 (PMC4620714; doi:10.1186/s12870-015-0639-6)
Supplement: Additional file 1: Table S1. — Codons under significant negative selection in PM susceptibility genes. Codon numbers refer to positions in the alignment of nine dicot MLO genes (AtMLO2, AtMLO6, AtMLO12, PsMLO1, MtMLO1, LjMLO1, CaMLO2, SlMLO1, NtMLO1) experimentally shown to act as powdery mildew susceptibility genes. Amino acid residues corresponding to each codon in barley HvMLO and pea PsMLO1 are indicated. For each residue, localization in any of the MLO protein domains, including seven transmembrane (TM) regions, three extracellular loops (E), three intracellular (I) loops, the N-terminus and the C-terminus, is indicated. Codons marked in bold are translated into class-specific residues. The threshold p-value was 0.1, representing the default value for Single-likelihood Ancestor Counting (SLAC) analysis implemented by the Datamonkey web server. (DOCX 30 kb) [file 12870_2015_639_MOESM1_ESM.docx]

**Additional file 3 (.docx): Table S1. Codons under significant negative selection in PM susceptibility genes.**

| **Codon** | **dN-dS** | **Normalized dN-dS** | **p-value** | **Residue in HvMLO** | **Residue in PsMLO1** | **Domain** |
| --- | --- | --- | --- | --- | --- | --- |
| 8 | -4.82 | -2.70 | 0.021 | A9 | E8 | N-terminus |
| 14 | -3.29 | -1.85 | 0.012 | T15 | T14 | TM1 |
| 16 | -4.12 | -2.31 | 0.004 | S17 | T16 | TM1 |
| 18 | -3.30 | -1.85 | 0.012 | A19 | A18 | TM1 |
| 20 | -3.30 | -1.85 | 0.012 | A21 | A20 | TM1 |
| 21 | -2.88 | -1.61 | 0.045 | V22 | V21 | TM1 |
| **24** | -2.60 | -1.46 | 0.091 | A25 | L24 | TM1 |
| **27** | -2.92 | -1.64 | 0.020 | V28 | L27 | TM1 |
| 30 | -2.47 | -1.38 | 0.044 | S31 | S30 | TM1 |
| **31** | -2.02 | -1.13 | 0.073 | V32 | I31 | TM1 |
| 49 | -4.87 | -2.73 | 0.007 | K50 | K49 | I1 |
| 55 | -2.47 | -1.38 | 0.037 | A56 | A55 | I1 |
| 58 | -3.16 | -1.77 | 0.037 | K59 | K58 | I1 |
| 62 | -4.83 | -2.71 | 0.006 | E63 | E62 | I1 |
| 65 | -3.49 | -1.96 | 0.012 | L66 | L65 | TM2 |
| 72 | -3.09 | -1.73 | 0.018 | L73 | L72 | TM2 |
| 73 | -2.93 | -1.64 | 0.025 | L74 | L73 | TM2 |
| 81 | -2.47 | -1.38 | 0.040 | A83 | S81 | E1 |
| 83 | -2.88 | -1.62 | 0.057 | I85 | I83 | E1 |
| 123 | -6.11 | -3.42 | 0.072 | / | D118 | E1 |
| 137 | -7.01 | -3.93 | 0.023 | / | R131 | E1 |
| 142 | -3.30 | -1.85 | 0.085 | / | K136 | E1 |
| 145 | -3.26 | -1.83 | 0.034 | D112 | D139 | E1 |
| 153 | -4.12 | -2.31 | 0.004 | V119 | V147 | E1 |
| 162 | -2.98 | -1.67 | 0.034 | H128 | H156 | E1 |
| 165 | -2.98 | -1.67 | 0.034 | H131 | H159 | TM3 |
| 166 | -2.80 | -1.57 | 0.038 | V132 | I160 | TM3 |
| 170 | -2.47 | -1.38 | 0.037 | V136 | V164 | TM3 |
| 171 | -2.71 | -1.52 | 0.029 | L137 | L165 | TM3 |
| 172 | -2.47 | -1.38 | 0.037 | A138 | A166 | TM3 |
| 176 | -3.94 | -2.21 | 0.014 | V142 | I170 | TM3 |
| **180** | -2.56 | -1.44 | 0.046 | V146 | I174 | TM3 |
| 186 | -2.47 | -1.38 | 0.044 | S152 | G180 | TM3 |
| 197 | -4.76 | -2.67 | 0.007 | E163 | E191 | I2 |
| 199 | -3.32 | -1.86 | 0.032 | E165 | E193 | I2 |
| 201 | -2.84 | -1.59 | 0.094 | T167 | R195 | I2 |
| **202** | -2.47 | -1.38 | 0.037 | S168 | T196 | I2 |
| 204 | -3.23 | -1.81 | 0.034 | E170 | E198 | I2 |
| 207 | -2.62 | -1.47 | 0.099 | F173 | F201 | I2 |
| 209 | -2.65 | -1.48 | 0.089 | N175 | N203 | I2 |
| 211 | -2.47 | -1.38 | 0.037 | P177 | P205 | I2 |
| 214 | -4.47 | -2.51 | 0.006 | F180 | F208 | I2 |
| 215 | -3.49 | -1.96 | 0.016 | R181 | R209 | I2 |
| **218** | -2.17 | -1.22 | 0.065 | H184 | R212 | I2 |
| **219** | -2.67 | -1.50 | 0.090 | Q185 | D213 | I2 |
| 222 | -2.98 | -1.67 | 0.034 | F188 | F216 | I2 |
| **224** | -4.09 | -2.30 | 0.007 | K190 | R218 | I2 |
| 225 | -2.28 | -1.28 | 0.063 | R191 | R219 | I2 |
| 226 | -2.98 | -1.67 | 0.034 | H192 | H220 | I2 |
| 233 | -3.63 | -2.03 | 0.030 | T198 | S227 | I2 |
| 234 | -3.55 | -1.98 | 0.052 | P199 | P228 | I2 |
| **237** | -2.62 | -1.47 | 0.036 | R202 | L231 | I2 |
| 239 | -2.24 | -1.25 | 0.060 | V204 | I233 | I2 |
| 243 | -2.98 | -1.67 | 0.034 | F208 | F237 | I2 |
| 244 | -3.47 | -1.94 | 0.017 | R209 | R238 | I2 |
| 247 | -2.64 | -1.48 | 0.090 | F212 | F241 | I2 |
| 250 | -3.79 | -2.13 | 0.016 | V215 | I244 | I2 |
| 254 | -2.98 | -1.67 | 0.034 | D219 | D248 | I2 |
| 258 | -2.94 | -1.65 | 0.022 | L223 | L252 | I2 |
| 259 | -2.26 | -1.27 | 0.069 | R224 | R253 | I2 |
| 261 | -2.47 | -1.38 | 0.040 | G226 | G255 | I2 |
| 262 | -2.98 | -1.67 | 0.034 | F227 | F256 | I2 |
| 265 | -3.29 | -1.85 | 0.012 | A230 | A259 | I2 |
| 270 | -2.77 | -1.55 | 0.097 | / | G264 | I2 |
| 280 | -2.98 | -1.67 | 0.047 | Y243 | Y274 | I2 |
| 281 | -3.05 | -1.71 | 0.019 | I244 | I275 | I2 |
| 288 | -4.47 | -2.51 | 0.006 | D251 | D282 | I2 |
| 293 | -3.29 | -1.85 | 0.012 | V256 | V287 | TM4 |
| 296 | -4.13 | -2.32 | 0.021 | S259 | S290 | TM4 |
| **297** | -2.47 | -1.38 | 0.037 | L260 | P291 | TM4 |
| 302 | -4.42 | -2.48 | 0.022 | V265 | F296 | TM4 |
| **304** | -2.47 | -1.38 | 0.037 | I267 | V298 | TM4 |
| **308** | -2.83 | -1.59 | 0.027 | F271 | L302 | TM4 |
| **309** | -2.88 | -1.61 | 0.045 | L272 | T303 | E2 |
| **321** | -2.47 | -1.38 | 0.037 | S284 | P315 | TM5 |
| 324 | -2.47 | -1.38 | 0.037 | P287 | P318 | TM5 |
| 325 | -2.78 | -1.56 | 0.099 | L288 | L319 | TM5 |
| **328** | -4.16 | -2.34 | 0.008 | L291 | I322 | TM5 |
| 329 | -2.95 | -1.65 | 0.030 | L292 | L323 | TM5 |
| 330 | -4.67 | -2.62 | 0.008 | C293 | L324 | TM5 |
| 331 | -2.47 | -1.38 | 0.037 | V294 | V325 | TM5 |
| 332 | -3.29 | -1.85 | 0.016 | G295 | G326 | TM5 |
| 334 | -3.35 | -1.88 | 0.033 | K297 | K328 | I3 |
| 338 | -2.36 | -1.32 | 0.054 | I301 | I332 | I3 |
| 342 | -5.60 | -3.136 | 0.010 | M305 | M336 | I3 |
| **343** | -2.47 | -1.38 | 0.046 | A306 | G337 | I3 |
| 348 | -4.27 | -2.40 | 0.042 | D311 | D342 | I3 |
| **350** | -2.47 | -1.38 | 0.045 | A313 | G344 | I3 |
| 351 | -2.69 | -1.51 | 0.088 | S314 | E345 | I3 |
| 357 | -3.29 | -1.85 | 0.012 | P320 | P351 | I3 |
| 360 | -2.79 | -1.57 | 0.096 | E323 | E354 | I3 |
| 361 | -4.13 | -2.32 | 0.019 | P324 | P355 | I3 |
| 366 | -2.98 | -1.67 | 0.034 | F329 | F360 | I3 |
| 368 | -4.47 | -2.51 | 0.006 | F331 | F362 | I3 |
| 375 | -2.45 | -1.38 | 0.038 | L338 | L369 | TM6 |
| 376 | -2.54 | -1.42 | 0.095 | F339 | F370 | TM6 |
| **381** | -3.29 | -1.85 | 0.012 | T344 | V375 | TM6 |
| 383 | -2.98 | -1.67 | 0.034 | F346 | F377 | TM6 |
| 386 | -2.47 | -1.38 | 0.037 | A349 | A380 | TM6 |
| 392 | -2.98 | -1.67 | 0.034 | F355 | F386 | TM6 |
| **401** | -2.98 | -1.67 | 0.034 | P363 | F393 | E3 |
| 408 | -2.98 | -1.67 | 0.033 | H370 | H400 | E3 |
| **413** | -4.47 | -2.51 | 0.006 | L375 | D405 | E3 |
| **416** | -4.37 | -2.45 | 0.004 | M377 | I408 | E3 |
| **417** | -2.33 | -1.31 | 0.065 | K378 | R409 | E3 |
| 424 | -3.20 | -1.79 | 0.067 | L385 | I416 | TM7 |
| 425 | -4.86 | -2.73 | 0.009 | Q386 | Q417 | TM7 |
| 430 | -2.98 | -1.67 | 0.047 | Y391 | Y422 | TM7 |
| **433** | -2.91 | -1.63 | 0.021 | F394 | L425 | TM7 |
| 435 | -3.19 | -1.79 | 0.014 | L396 | L427 | TM7 |
| 438 | -2.01 | -1.12 | 0.076 | L399 | L430 | TM7 |
| 443 | -2.47 | -1.38 | 0.038 | G404 | G435 | C-terminus |
| 447 | -3.92 | -2.20 | 0.054 | K408 | K439 | C-terminus |
| **448** | -2.47 | -1.38 | 0.037 | R409 | P440 | C-terminus |
| 450 | -2.74 | -1.54 | 0.059 | I411 | I442 | C-terminus |
| 451 | -2.98 | -1.67 | 0.034 | F412 | F443 | C-terminus |
| 458 | -2.47 | -1.38 | 0.037 | A419 | A450 | C-terminus |
| 459 | -3.49 | -1.96 | 0.012 | L420 | L451 | C-terminus |
| 466 | -3.29 | -1.85 | 0.012 | A427 | A458 | C-terminus |
| **468** | -2.86 | -1.61 | 0.093 | E429 | K460 | C-terminus |
| 472 | -4.14 | -2.32 | 0.022 | V433 | Q464 | C-terminus |
| **476** | -3.29 | -1.85 | 0.022 | D437 | S468 | C-terminus |
| 483 | -2.47 | -1.38 | 0.037 | P449 | P473 | C-terminus |
| **487** | -5.72 | -3.21 | 0.001 | S453 | R477 | C-terminus |
| 496 | -3.29 | -1.85 | 0.013 | S462 | S486 | C-terminus |
| 497 | -4.12 | -2.31 | 0.004 | P463 | P487 | C-terminus |
| 499 | -2.98 | -1.67 | 0.034 | H465 | H489 | C-terminus |
| 500 | -3.36 | -1.89 | 0.013 | L466 | L490 | C-terminus |
| 501 | -3.59 | -2.01 | 0.023 | L467 | L491 | C-terminus |
| 502 | -2.98 | -1.67 | 0.077 | H468 | H492 | C-terminus |
