# Supplementary figures and images for: Monocot and dicot MLO powdery mildew susceptibility factors are functionally conserved in spite of the evolution of class-specific molecular features
Source: BMC Plant Biol. 2015 Oct 26;15:257. doi: 10.1186/s12870-015-0639-6 (PMC4620714; doi:10.1186/s12870-015-0639-6)

a)

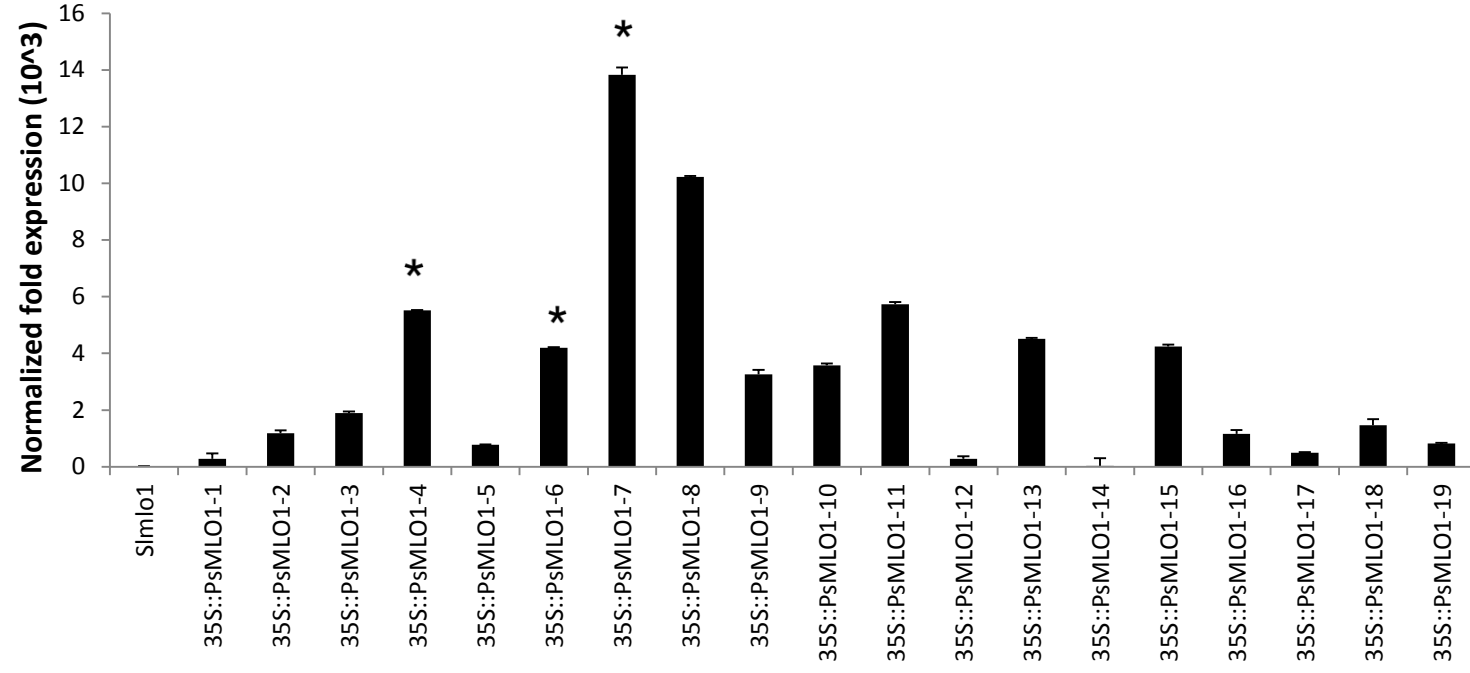

b)

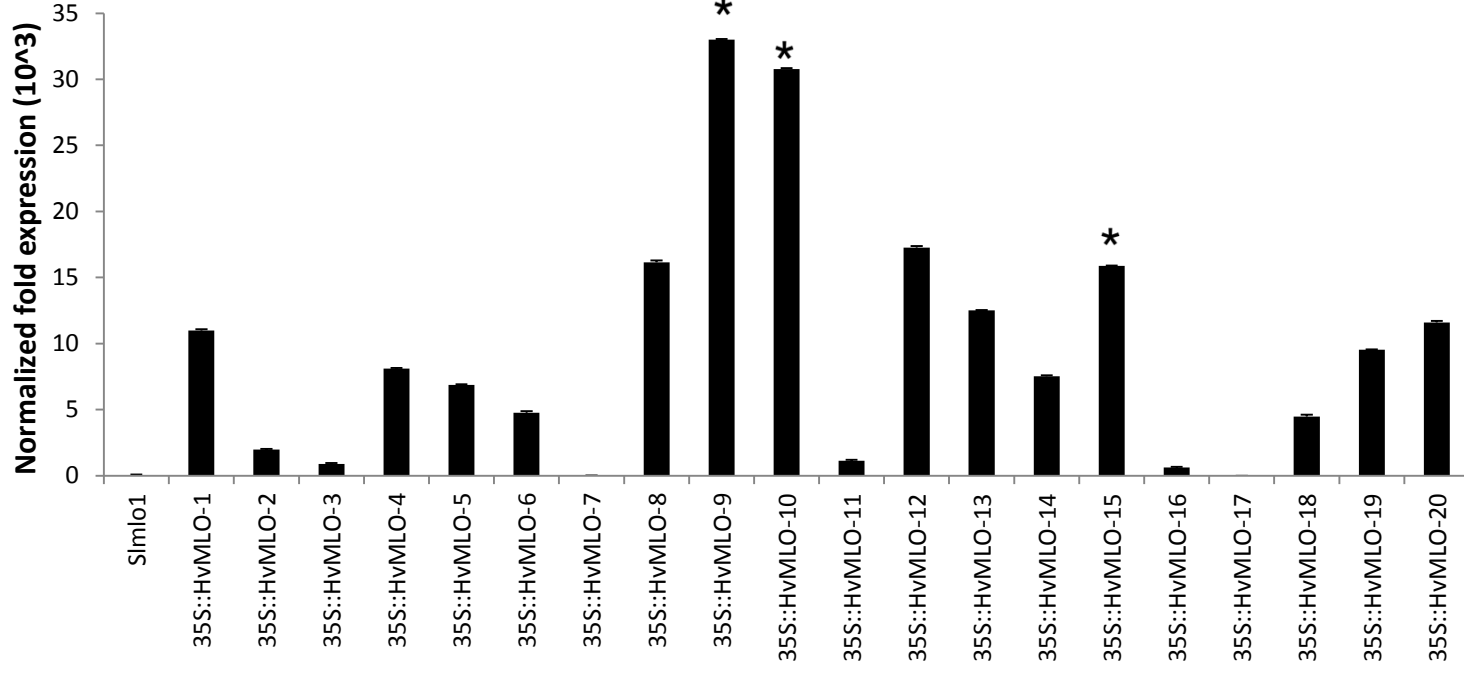

Supplement: Additional file 2: Figure S1. — Expression levels of PsMLO1 and HvMLO after transformation. Panel A) and panel B) show the expression of PsMLO1 and HvMLO in 19 and 20 T1 individuals, respectively, which were obtained by the transformation of the tomato mutant line Slmlo1, harboring a loss-of-function mutation of the endogenous SlMLO1 gene. Asterisks indicate T1 individuals selected for self-pollination and the development of T2 families. (PDF 173 kb) [file 12870_2015_639_MOESM2_ESM.pdf]

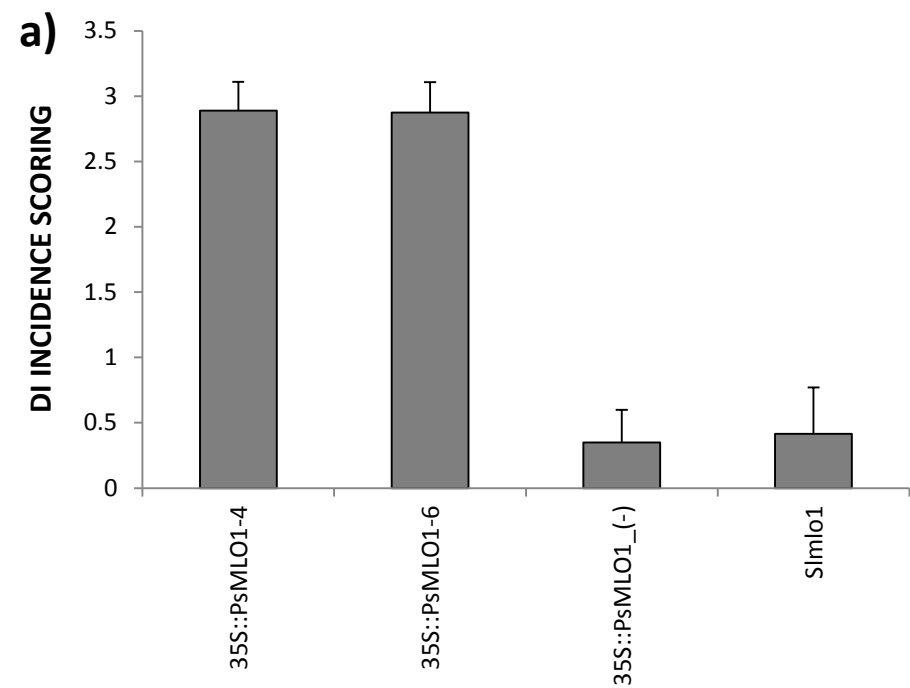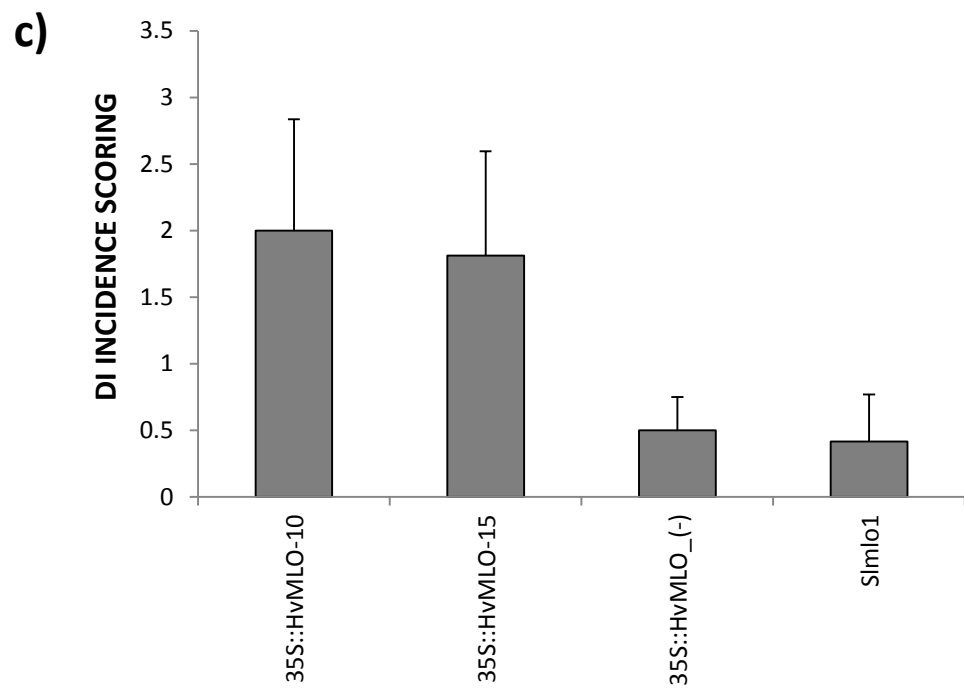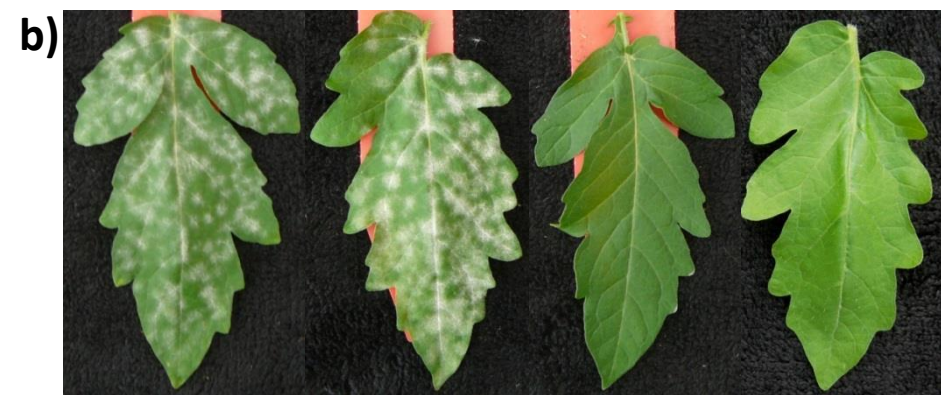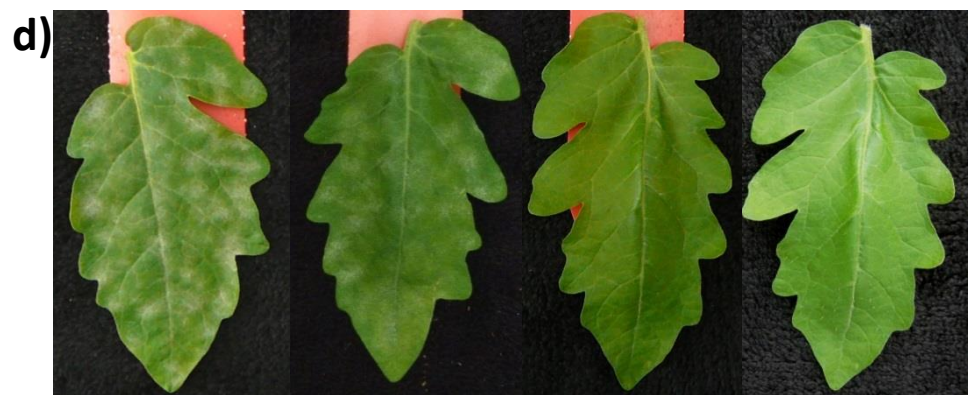

Supplement: Additional file 3: Figure S2. — Effects of transgenic overexpression of pea PsMLO1 and barley HvMLO in the tomato mutant line Slmlo1. Average disease index (DI) values and phenotypes are referred to transgenic plants of two additional T2 families segregating for PsMLO1 [35S::PsMLO1-4 and 35S::PsMLO1-6, panel a) and b)] and two additional T2 families segregating for HvMLO [35S::HvMLO-10 and 35S::HvMLO-15), panel c) and d)]. Data relative to the Slmlo1 mutant line, used as genetic background for transformation, and non-transgenic plants of three T2 families for each overexpression construct (35S::PsMLO1_(−) and 35S::HvMLO_(−)) are also shown. (PDF 304 kb) [file 12870_2015_639_MOESM3_ESM.pdf]
