# Supplementary material for: Monocot and dicot MLO powdery mildew susceptibility factors are functionally conserved in spite of the evolution of class-specific molecular features
Source: BMC Plant Biol. 2015 Oct 26;15:257. doi: 10.1186/s12870-015-0639-6 (PMC4620714; doi:10.1186/s12870-015-0639-6)
Supplement: Additional file 4: Table S2. — Primer pairs used in this study. (DOCX 14 kb) [file 12870_2015_639_MOESM4_ESM.docx]

**Additional file 4 (.docx): Table S2.** **Primer pairs used in this study**.

| **Name** | **Fw primer sequence (5'--> 3')** | **Rev primer sequence (5'-->3')** |
| --- | --- | --- |
| PsMLO1 | CACCATGGCTGAAGAGGGAGTTAAGGAAC | CTAATTGCTCCCTAAGTGGCGCTT |
| HvMLO | CACCGACCGATGTCGGACAAAA | TCATCCCTGGCTGAAGGAAAA |
| qPsMLO1 | AGGTTTGCAAGGGACACAAC | TTGTGCATCATGTCCTGGAG |
| qHvMLO | TTTCATCCCTCTCGTGATCC | CCACTGTCCACACAAAATGC |
| NPTII | TCGGCTATGACTGGGCACAAC | AAGAAGGCGATAGAAGGCGA |
| 35S | GCTCCTACAAATGCCATCA | GATAGTGGGATTGTGCGTCA |
| Oid | CGCCAAAGACCTAACCAAAA | AGCCAAGAGATCCGTTGTTG |
| qEf | ACAGGCGTTCAGGTAAGGAA | GAGGGTATTCAGCAAAGGTCTC |
